# Ross River Virus Seroprevalence, French Polynesia, 2014–2015

**DOI:** 10.3201/eid2310.170583

**Published:** 2017-10

**Authors:** Maite Aubry, Anita Teissier, Michael Huart, Sébastien Merceron, Jessica Vanhomwegen, Claudine Roche, Anne-Laure Vial, Sylvianne Teururai, Sébastien Sicard, Sylvie Paulous, Philippe Desprès, Jean-Claude Manuguerra, Henri-Pierre Mallet, Didier Musso, Xavier Deparis, Van-Mai Cao-Lormeau

**Affiliations:** Institut Louis Malardé, Tahiti, French Polynesia (M. Aubry, A. Teissier, C. Roche, S. Teururai, D. Musso, V.-M. Cao-Lormeau);; Centre D’épidémiologie et de Santé Publique des Armées, Marseille, France and Unité Mixte de Recherche Sciences Economiques et Sociales de la Santé et Traitement de l’Information Médicale, Marseille (M. Huart, S. Sicard, X. Deparis);; Institut de la Statistique de la Polynésie Française, Tahiti and Institut National de la Statistique et des Études Économiques, Sainte Clotilde, Réunion (S. Merceron);; Institut Pasteur, Paris, France (J. Vanhomwegen, S. Paulous, J.-C. Manuguerra);; Direction Départementale de la Cohésion Sociale et de la Protec-tion des Populations, Yonne, France (A.-L. Vial);; Direction de la Santé de la Polynésie Française, Tahiti (A.-L. Vial, H.-P. Mallet);; Université de La Réunion, Sainte Clotilde, France;; Unité Mixte de Recherche Processus Infectieux en Milieu Insulaire Tropical, Sainte Clotilde (P. Desprès)

**Keywords:** Ross River, Arbovirus, French Polynesia, Pacific, seroprevalence, immunoglobulin G

## Abstract

Ross River virus (RRV), spread by *Aedes* and *Culex* mosquitoes, is the most commonly transmitted arbovirus in Australia. A serosurvey of blood donors in French Polynesia during 2011–2013 suggested that RRV circulated without being detected. We report RRV circulation in French Polynesia based on further screening of blood samples collected during 2014–2015.

Ross River virus (RRV), an alphavirus of the family *Togaviridae*, is an arbovirus transmitted by *Aedes* and *Culex* mosquito species (*1*). Symptoms of RRV infections mainly consist of fever, arthralgia, and rash. RRV was first isolated in North Queensland in 1959 and has become the most common arboviral disease in Australia (*2*). RRV outbreaks were reported during 1979–1980 in Pacific Island countries and territories (PICTs) including Fiji, Cook Islands, American Samoa, and New Caledonia (*1*). 

In French Polynesia (FP), a territory of the Pacific region with ≈270,000 inhabitants, dengue virus (DENV) was the only arbovirus detected until identification of the Zika virus (ZIKV), then chikungunya virus (CHIKV), causing outbreaks during 2013–2014 and 2014–2015, respectively (*3*). Although RRV infections have never been reported in FP, a serosurvey of blood donors during 2011–2013 suggested that RRV has circulated without being detected (*4*). In this study, we report additional evidence of RRV circulation in FP based on further screening of blood samples collected during 2014–2015 and previously used for a ZIKV serosurvey (*5*).

We tested 3 groups of participants randomly sampled in FP for the presence of anti-RRV IgG by using recombinant antigen-based indirect ELISA (patent no.WO2012076715A1) and microsphere multiplex immunoassay (patent no. WO2013083847A2) (*4*,*5*). The first group consisted of 196 participants recruited during February–March 2014 in 8 of the most inhabited islands of the 5 FP archipelagos (archipelagos listed in parentheses): Tahiti and Moorea (Society), Rangiroa and Makemo (Tuamotu), Nuku Hiva and Hiva Oa (Marquesas), Rurutu (Austral), and Rikitea (Gambier). The second group included 700 participants recruited during September–November 2015 on the 2 most inhabited islands of FP: Tahiti and Moorea. The third group consisted of 476 schoolchildren recruited during May–June 2014 on Tahiti. The Ethics Committee of French Polynesia approved recruitment of participants and processing of blood samples (approval no. 60/CEPF-06/27/2013). We analyzed seroprevalence data by using the Fisher exact test, and considered p values >0.05 as statistically significant.

RRV seropositivity rates among participants sampled in the 5 archipelagos in 2014 ranged 16% to 49% (average 35%), and were significantly different between the Society and Marquesas (p = 0.036), Tuamotu and Marquesas (p = 0.001), and Tuamotu and Austral-Gambier (p = 0.002) Islands (Table). In the Society Islands, screening of additional participants in 2015 did not lead to a significant difference in RRV seroprevalence (18%) compared with the 1 observed in the participants recruited in 2014 (27%) (p = 0.125). In contrast, RRV seroprevalence among schoolchildren (1%) was significantly lower than in the general population from the Society Islands, during both 2014 and 2015 (p<0.0001). We compared 2 groups in the general population in 2014 and 2015. Rates of participants whose samples were RRV-seropositive and who were born or arrived in FP before 1982 (respectively, 39% and 19%) and thus potentially exposed to the last reported epidemic in the Pacific, were not significantly different from those who were born or arrived during or after 1982 (respectively, 28% and 15%).

**Table:** Seropositivity for Ross River virus among participants randomly recruited in French Polynesia from the general population during February–March, 2014 and September–November, 2015; and from schoolchildren during May–June 2014.

Sampled population, time of sampling, location of sampling	Median age (range), y	No. seropositive/No. born or arrived in French Polynesia before 1982 (% [95% CI]*)	No. seropositive/No. born or arrived in French Polynesia from 1982 (% [95% CI])	Total no. seropositive/Total no. tested (% [95% CI])
General population				
February-March 2014				
Society Islands	47 (13–77)	9/29 (31 [14–48])	4/20 (20 [11–40 ])	13/49 (27 [9–45])
Tuamotu Islands	39 (7–86)	6/28 (21 [6–37])	2/21 (10 [4–27])	8/49 (16 [6–26])
Marquesas Islands	45 (10–82)	15/32 (47 [30–64])	9/17 (53 [29–77])	24/49 (49 [36–62])
Austral-Gambier Islands	38 (7–84)	15/26 (58 [39–77])	8/23 (35 [15–54])	23/49 (47 [35–59])
Total	41 (7–86)	45/115 (39 [30–48])	23/81 (2 [19–38])	68/196 (35 [27–43])
September-November 2015				
Society Islands	43 (4–88)	77/397 (19 [16–23])	46/303 (15[11–19])	123/700 (18 [15–21])
Schoolchildren				
May-June 2014				
Society Islands	11 (6–16)	−	6/476 (1[0–2])	6/476 (1 [0–2])

*Confidence intervals (CI) were calculated by using Fischer’s exact test.

Although no RRV outbreaks were reported in the PICTs after 1980, identification of RRV infections among travelers returning from Fiji between 1997–2009 suggested subsequent circulation of the virus in the Pacific (*6*,*7*). In a serosurvey conducted in American Samoa in 2010, the finding that 63% of participants born after 1980 and who had lived their whole lives in the territory were seropositive for RRV also supported this assumption (*8*). In our study, detection of RRV-seropositive participants who were born or arrived in FP from 1982 shows that the virus probably circulated after the end of the 1979–1980 epidemic in the Pacific. This finding corroborates data previously obtained regarding blood donors (*4*).

In this study, we also provide evidence that RRV circulated in all archipelagos. The overall seroprevalence rate of 35% found in the general population in 2014 is consistent with the rate of 34.40% previously obtained in blood donors (*4*), with both groups of participants mainly including adults (median 41 and 36 years, respectively). The lower seroprevalence found in schoolchildren (median 11 years) compared with the general population in 2015 (median 43 years) is consistent with previous age-stratified studies showing that RRV seroprevalence increases with age (*1*). Nevertheless, because the seroprevalence observed in children is lower in FP than in endemic Australian areas (*9*), another reasonable explanation is that RRV circulated poorly in FP during the 16 years before the study. This observation is also supported by the small number of seropositive participants who arrived or were born after 2000 in FP, among the general population in 2014 (3/18) and 2015 (0/54) (data not shown).

Increasing evidence that RRV circulated silently in several PICTs, in the absence of marsupial animal reservoirs (*8*), supports the need for enhanced laboratory and epidemiologic surveillance. Moreover, clinicians should be aware of the potential for RRV infections to occur. As illustrated with ZIKV and CHIKV, tropical islands are new hubs for emerging arboviruses and neither diseases nor places should be neglected (*10*).
